# Deep Sequencing of Suppression Subtractive Hybridisation Drought and Recovery Libraries of the Non-model Crop *Trifolium repens* L.

**DOI:** 10.3389/fpls.2017.00213

**Published:** 2017-02-23

**Authors:** Maciej Bisaga, Matthew Lowe, Matthew Hegarty, Michael Abberton, Adriana Ravagnani

**Affiliations:** Institute of Biological, Environmental and Rural Sciences, Aberystwyth UniversityAberystwyth, UK

**Keywords:** white clover, drought, recovery, SSH libraries, MiSeq

## Abstract

White clover is a short-lived perennial whose persistence is greatly affected by abiotic stresses, particularly drought. The aim of this work was to characterize its molecular response to water deficit and recovery following re-hydration to identify targets for the breeding of tolerant varieties. We created a white clover reference transcriptome of 16,193 contigs by deep sequencing (mean base coverage 387x) four Suppression Subtractive Hybridization (SSH) libraries (a forward and a reverse library for each treatment) constructed from young leaf tissue of white clover at the onset of the response to drought and recovery. Reads from individual libraries were then mapped to the reference transcriptome and processed comparing expression level data. The pipeline generated four robust sets of transcripts induced and repressed in the leaves of plants subjected to water deficit stress (6,937 and 3,142, respectively) and following re-hydration (6,695 and 4,897, respectively). Semi-quantitative polymerase chain reaction was used to verify the expression pattern of 16 genes. The differentially expressed transcripts were functionally annotated and mapped to biological processes and pathways. In agreement with similar studies in other crops, the majority of transcripts up-regulated in response to drought belonged to metabolic processes, such as amino acid, carbohydrate, and lipid metabolism, while transcripts involved in photosynthesis, such as components of the photosystem and the biosynthesis of photosynthetic pigments, were up-regulated during recovery. The data also highlighted the role of raffinose family oligosaccharides (RFOs) and the possible delayed response of the flavonoid pathways in the initial response of white clover to water withdrawal. The work presented in this paper is to our knowledge the first large scale molecular analysis of the white clover response to drought stress and re-hydration. The data generated provide a valuable genomic resource for marker discovery and ultimately for the improvement of white clover.

## Introduction

White clover (*Trifolium repens L*.) is an important component of sustainable agricultural systems, because of its high nutritional value and the ability of fixing atmospheric nitrogen. It is grown in temperate regions, mainly in mixed swards with companion ryegrass. White clover propagates and persists primarily by stolons. As the plant grows, the stolons break generating a large number of smaller plants. At this stage, the plants become more vulnerable to biotic and abiotic stresses (Brock et al., 1988; Fothergill et al., 1997; Sanderson et al., 2003) and the white clover component within a sward can undergo a dramatic decline, the so called “clover crash” (Fothergill et al., 1996). This phenomenon makes the crop performance unreliable and therefore less attractive to farmers, as the crop benefits are of little use, unless coupled with good persistence.

Water deficiency is one of the environmental factors most effecting crop productivity and its negative effects on white clover are well documented. A 30 year study in Australia showed that water availability was the main limiting factor in white clover persistence (Hutchinson et al., 1995), while Belaygue and colleagues observed an 80% decrease in stolon numbers as a result of just a 30% decrease in relative water content in five different genotypes of white clover (Belaygue et al., 1996).

White clover is recognized to be not as adapted to drought conditions as other forage legumes, such as lucerne. This is mainly due to its shallow rooted system and its poor control of stomata closure (Hart, 1987). Additionally, very limited variability in terms of drought tolerance has been found amongst the white clover genotypes available (Abberton and Marshall, 2005), hence the need to develop molecular tools that make the identification of source of variation more efficient than methods based entirely on phenotypic characterization (Prohens, 2011). The development of genomic resources for forage legumes has been slower than for grain legumes and other crops because of the complexity of their genome, heterozygosity and polyploidy (Annicchiarico et al., 2015). However, the number of transcription profiling is steadily growing and is available not only for model species, such as *Medicago truncatula*, but also for legume crops, such as soybean, chickpea, pigeonpea (Chen et al., 2008; Pandey et al., 2016 and references therein). In particular, transcriptome profiling of red clover under prolonged drought stress has recently become available (Yates et al., 2014). This work has provided useful insights in the response to drought of a crop phylogenetically close to white clover. However, red and white clovers are quite different in their growth habits and there was a need for investigating white clover response at an early stage of droughting, when the crop is most affected (Fothergill et al., 1996).

Several approaches are available for studying transcription profiling from early methods, such as Expressed Sequence Tags (EST) and Suppression Subtractive Hybridization (SSH), through to microarray technology and more recently Next Generation Sequencing (NGS) platforms (Deshmukh et al., 2014). SSH is a powerful technique for the amplification of differentially expressed genes by simultaneous amplification and suppression of target and non-target sequences, respectively (Diatchenko et al., 1996). Despite the advent of next generation sequencing, SSH is still a popular technique because of the relatively low cost, low amount of starting material and the relatively low rate of false positives. It is however quite laborious and costly if the number of sequenced clones is increased to several thousands.

We have revisited four SSH libraries constructed from leaf tissue of white clover plants subjected to and recovering from drought stress (Bisaga, 2013). In this work, only the forward drought and recovery libraries were sequenced, yielding 1,127 and 2,405 unigenes, respectively. By replacing the cloning step with Illumina technology sequencing, we were able to exploit the already available SSH libraries more efficiently by sequencing both forward and reverse libraries at a deep coverage (387x). This allowed us to identify 6,937 and 3,142 transcripts up- and down-regulated, respectively, in response to drought, and 6,695 and 4,897, in response to re-hydration, providing a global picture of transcriptional changes occurring in white clover under the two treatments.

Most published studies analyze the effects of water deprivation on white clover from an agronomic and physiological point of view, but no studies are available in which the effect of water deprivation is characterized at a molecular level. This work is, to our knowledge, the first report of a large scale molecular study on the response of white clover to drought and re-hydration.

## Materials and methods

### Plant material and growth conditions

The white clover (*T. repens L*.) R3R4 genotype, isolated at IBERS for research purposes, was used in this study (Febrer et al., 2007).

Forty eight clonal cuttings of R3R4 were grown in 3.5 inch pots filled with a John Innes #3 potting soil mix (JI#3 Base: sterilized loam: peat: grit: fertilizer = 1:1:1:1 by volume). The plants were grown at 20/10°C (day/night) under daylight with additional illumination provided by high pressure sodium lamps (Philips: 400 watt Son-T Agro) to give a photoperiod of 10 h, under glasshouse condition. After root formation, clones were transferred into 5 inch pots and grown for 5 weeks. Following re-potting into 8 inch pots, the plants were grown for further 6 weeks before applying the stress treatment.

### Stress treatment and measurements

The plants were organized in four complete blocks. Each experimental block consisted of nine water-stressed and three watered control plants. Before dehydration treatment, plants were watered to full capacity (ca. 500 ml). Under our experimental condition the average water loss was ca. 200 ml/day (estimated by weighing the pots; Image 1 of Supplemental Data). This volume of water was added to the control plants daily to maintain them at a uniform non-stressed water status, while the plants nominated for droughting were subject to 9 days of restricted water regime (decreasing from 100 ml to nil), followed by three recovering days in which they were watered to full capacity again. Leaf material was collected daily between 8.00 am and 9.00 am, immediately frozen in liquid nitrogen and stored at −80°C for RNA extraction.

Two young fully expanded leaves were used for relative water content (RWC) measurement. Leaves were weighed immediately after collection (fresh weight–FW), placed in water to hydrate to full turgidity and weighed again (turgid weight–TW). Samples were then dried overnight at 80°C and weighed (dry weight–DW). The RWC was calculated according to Smart and Bingham equation (Smart and Bingham, 1974): RWC = [(FW–DW)/(RW–DW)] × 100.

### RNA extraction, cDNA synthesis and sqRT-PCR

Total RNA was extracted from 0.2 grams of leaf tissue from day1, 4, and 10 using an RNeasy® Plant Mini Kit (Qiagen) according to the manufacturer's instructions. cDNA was synthesized from 1 μg of total RNA using Oligo (dT)25 primer (0.5 mM final) and 200 U of RevertAid™ H Minus M-MuLV Reverse Transcriptase (Fermentas) in 20 μl reaction, according to manufacturer's instructions.

All primers used for sqRT-PCR were designed using Roche Universal Probe Library Assay Design Software (https://lifescience.roche.com/webapp/wcs/stores/servlet/CategoryDisplay?tab=Assay+Design+Center&identifier=Universal+Probe+Library&langId=-1) and are listed in Table S1.

The *NCED* gene was amplified from *T. repens* using a pair of degenerate primers (NCED5 and NCED3) designed on publicly available nucleotide sequences (Acc. No.: FF391502.1, FF393344.1, BB931293.1, BB929043.1, 5g025250.1, 5g025270.1, and 5g025290.1).

Primers NCEDmb2F and NCEDmb2R suitable for sqRT-PCR were designed on the newly sequenced *T. repens NCED* gene. The housekeeping actin gene used as a control was amplified using primers TrACT5 and TrACT3 designed on the publicly available *T. repens* gene (Acc. No. AM419900.1).

sqRT-PCR was performed in triplicate using Power SYBR® Green PCR Master Mix (Applied Biosystems) on a LightCycler® 480 Real-Time Instrument (Roche) according to manufacturer's instruction. A 10 μl reaction containing 100 ng of cDNA template and 100 nM of each gene-specific primer was subject to 40 cycles of 15 s at 95°C, 1 min at 57°C and 40 s at 72°C. PCR products were subjected to dissociation curve analysis by incubating at 15 s at 95°C, 15 s at 60°C, 15 s at (60°–5°C) and data were analyzed using the the 2^−ΔΔCT^ method to quantify relative transcript abundance (Livak and Schmittgen, 2001). For internal control, the housekeeping actin gene was amplified from cDNA of control, drought and recovery and samples.

### Construction of subtractive libraries

Four Suppression Subtractive Hybridisation (SSH) libraries were generated using the SMARTer™ PCR cDNA Synthesis Kit (Clontech) and the PCR-Select cDNA Subtraction Kit (Clontech) according to manufacturer's instructions. Two libraries were constructed by subtracting driver RNA sampled from plants at day 0 of the experiment from tester RNA sampled from stressed plants at day 4 of the drought treatment (Drought Forward library–DF) and vice-versa (Drought Reverse library–DR). The other two libraries were constructed by subtracting the same driver RNA at day 0 from tester RNA sampled from plants at day 1 of the rehydration process (Recovery Forward library - RF) and vice-versa (Recovery Reverse library–RR). Products of the secondary hybridisations were re-amplified using Nested PCR primer 1 and R2 (from PCR-Select cDNA Subtraction Kit; Clontech), purified using QIAquick PCR Purification Kit (Qiagen) and digested with RsaI to remove the adaptors prior to the library preparation for sequencing.

### Sequencing and data analysis

RsaI digested samples were purified with AMpure XP beads (Agencourt) according to manufacturer's instructions and used for the construction of indexed sequencing libraries using the Illumina Nextera XT sample preparation kit as per the manufacturer's instructions. Libraries were pooled and diluted to a final concentration of 6 pM prior to sequencing on two independent runs using the Illumina MiSeq platform.

*De novo* assembly and read mappings were carried out using CLC Genomics Workbench 5.5.2 (CLC bio A/S, Aarhus, Denmark).

Statistical comparison of libraries was carried out using SAGEstat V4.2 (Ruijter et al., 2002) and *q*-value for false discovery rate was estimated using the R package *q*-value V1.0 (Storey, 2002).

Functional annotation was carried out using Blast2GO 2.8.0 (Conesa et al., 2005). Statistical analysis of KEGG pathways was carried out using EC2KEGG (Porollo, 2014) and full results are shown in Table S5.

MapMan analysis was carried out using MapMan 3.5.1R2 (Thimm et al., 2004) and Mercator (Lohse et al., 2014).

### Data availability

Next Generation Sequencing (NGS) runs have been deposited at National Centre for Biotechnology Information (NCBI) in the Short Read Archive (SRA) database under accessions numbers SRR3621208, SRR3621729, SRR3621829 and SRR3621860. Contig sequences have been deposited in the Transcriptome Shotgun Assembly (TSA) database GEST00000000. The version described in this paper is the first version, GEST01000000. Since TSA does not accept sequences shorter than 200 bp and containing a stretch of “N” > 14 nucleotides, the complete set of assembled sequences is provided as Data Sheet 1 of Supplemental Data.

## Results and discussion

### Fast drought experiment

**Figure 1:**
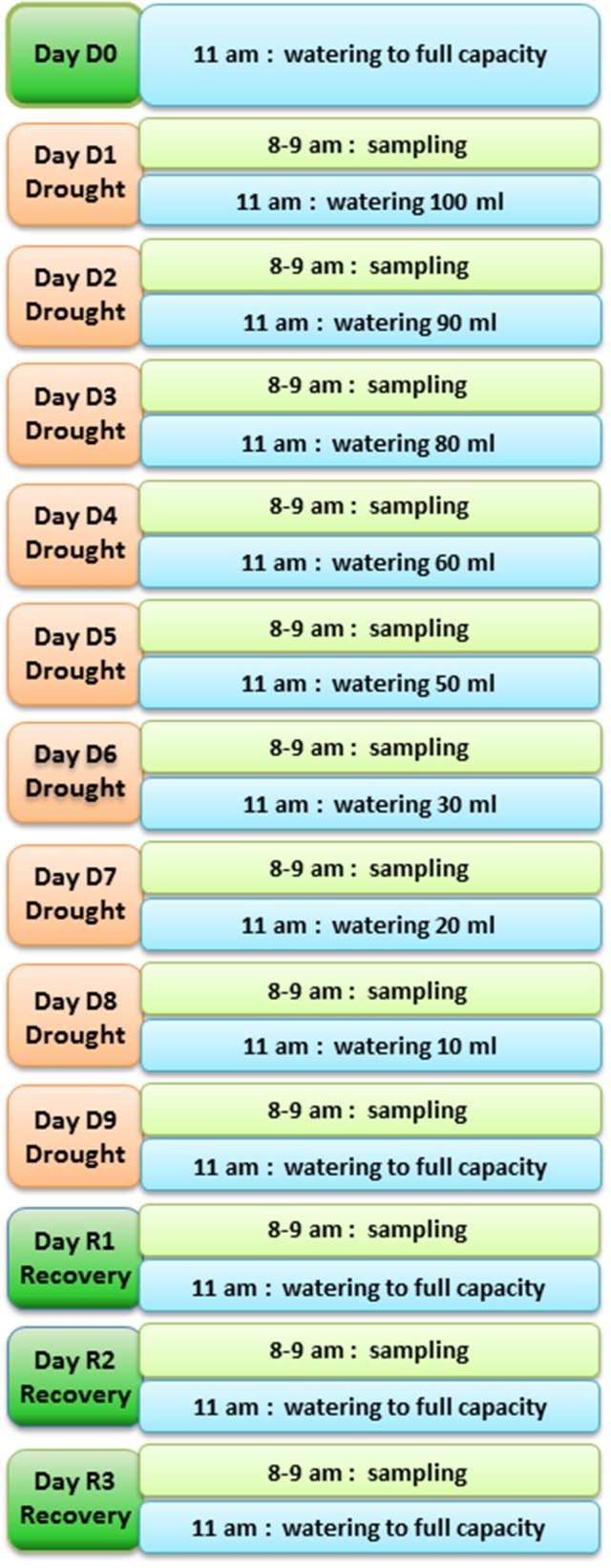
**Diagram of the fast drought experiment set up**. Time of sampling and time and volume of watering are shown for each day of the experiment.

**Figure 2:**
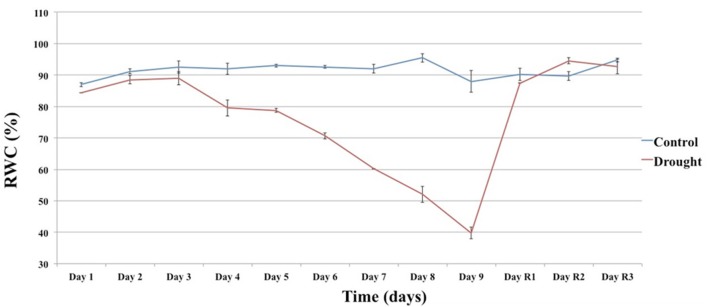
**The effect of the drought (Day 1 to Day 9) and recovery (Day R1 to R3) treatment on RWC**. Each value is the mean of two technical replicates. Error bars denote standard error.

To identify genes showing changes in expression in response to water deficit and subsequent re-hydration, 48 plants of *T. repens* (genotype R_3_R_4_; Febrer et al., 2007) were subjected to a fast drought experiment as described in Material and Methods. The conditions were chosen following a number of pilot studies that tested pot size, soil volume, watering regime, and sampling technique/timing. The amount of water applied during the experiment decreased daily as shown in Figure 1. The leaf relative water content (RWC) was measured every day of the experiment to monitor the stress status of the plants. Plants whose RWC consistently declined every day and showed a rapid response to re-watering were selected for the RNA extraction. Figure 2 shows the RWC of the stressed plants in comparison to the control. The first significant decline in RWC is observed at day 4 (9.5% drop compared to un-stressed plants) and it reverted back to the same level as the control plants within 24 h of restoring normal watering (day 1 of recovery treatment).

The stress status of the experimental plants was also monitored at molecular level in semi-quantitative Real Time PCR (sqRT-PCR) using the 9-cis-epoxycarotenoid dioxygenase (*NCED*) gene and the actin gene as internal control. The product of *NCED* gene catalyzes the first step of ABA biosynthesis from carotenoids in chloroplasts and it is therefore activated very quickly in response water deficit stress, making it a useful marker for monitoring the onset of drought response (Iuchi et al., 2000).

**Figure 3:**
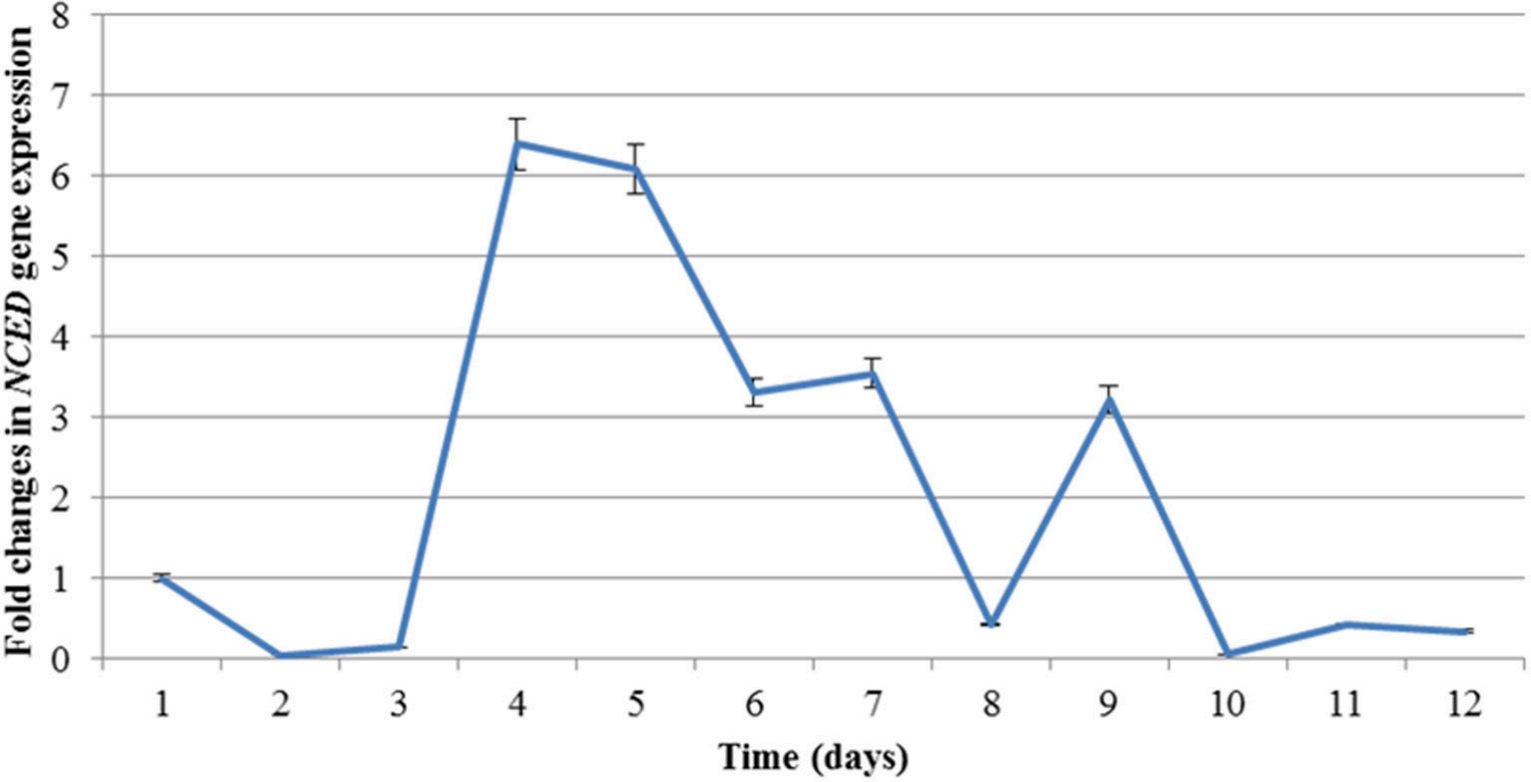
**Expression profile of *NCED* during drought (Day 1 to Day 9) and recovery (Day R1 to R3) treatment analysed by RT-PCR**. Error bars denote standard deviation.

Figure 3 shows the expression profile of the *NCED* transcript during the course of the experiment. The level of expression increases sharply between day 3 and 4 (~6.4-folds). The expression starts decreasing after day 5 and, after a brief increase on day 9, returns to basal level following re-watering (day 9). This expression profile seems to be typical of *NCED*, as also observed in *M. truncatula* under drought stress (probe Mtr.35044.1.S1_at of the microarray experiment by Zhang et al. unpublished at http://mtgea.noble.org/v3/probeset.php?id=Mtr.35044.1.S1_at).

The concomitant decrease in RWC and increase in *NCED* expression was a good indication of the beginning of the stress response.

### Construction and sequencing of SSH libraries

Based on the RWC monitoring and the *NCED* expression time course, plant material from day 4 (Tester D4) and day 10 (Tester R1–day 1 of the recovery treatment) were chosen for the library preparation. In both cases, the plant material from day 1 was used as control (Driver D1).

Four SSH libraries were constructed. For the drought treatment a forward library (DF) was constructed by using the RNA from D4 plant material as tester and the one from D1 as driver and a reverse library (DR) using D1 RNA as tester and D4 as driver. Similarly, for the recovery treatment, a forward (RF) library was constructed subtracting D1 RNA from R1 RNA and a reverse (RR) subtracting R1 RNA from D1 RNA.

The libraries were constructed according to the Diatchenko‘s method (Diatchenko et al., 1996). In the original method, the libraries were cloned into *E. coli* and the individual clones are then Sanger sequenced. This laborious procedure was originally employed to sequence the drought forward library (Bisaga, 2013). The availability of next generation sequencing technology can however speed up the procedure and generate a much greater amount of data at a fraction of the cost per clone. We therefore omitted the cloning step and sequenced the products of the secondary amplification of the four libraries directly using the Illumina MiSeq platform.

### *De novo* and reference-based assembly of a reference transcriptome

The *T. repens* genome is not yet available, however, at the time this work was carried out, a transcriptome sequence (Nagy et al., 2013) and the genome of the closely related *M. truncatula* were publicly available (http://www.jcvi.org/medicago/display.php?pageName=General&section=Download). Neither resource was on its own optimal as a reference sequence to aid the reads assembly. The published transcriptome of 71,545 sequences was generated by *de novo* assembly of 454-pyrosequencing reads. It was obtained from a mixture of above ground tissues of white clover grown under un-stressed conditions, while ours was generated from stressed plants and, like all transcriptomes, could contain mis-assembled and chimeric transcripts. For *M. truncatula*, the whole transcript set is available from the genome annotation and the average similarity between *T. repens* and *M. truncatula* at transcript level is around 90%. However, when the white clover transcriptome is BLASTed against the *Medicago* CDS library (Mt4.0v1), using a cutoff *E*-value of 10^−20^, only 60% of the sequences had hits. We therefore decided to use a combination of *de novo* and reference-based assembly.

Table 1 shows the number of reads generated for each library before and after quality checks. DR and RF libraries generated a larger number of reads because they were run with an updated MiSeq system. Reads were trimmed for quality (quality limit = 0.01), length (≥36 bp) and for Nextera and SSH adaptors using CLC Genome Workbench 5.5.2. Reads from all four libraries (50,756,845 in total) were *de novo* assembled into 20,981 contigs of an average length of 482 bp (word size = 24; bubble size 111; minimum contig length = 200; mismatch cost = 2; insertion cost = 3; length fraction = 0.5; similarity fraction = 0.9).

**Table 1:** **Summary of quality and adaptor trimming**.

	**DF**	**DR**	**RF**	**RR**
No. paired reads	5,801,548	20,157,262	23,573,618	6,324,432
Average length	151	123	131	151
No. paired reads after trimming	4,905,542	17,645,418	20,549,048	5,360,350
% trimmed	85	88	87	85
Average length	105	110	115	108
No. broken pairs	228,878	750,518	1,066,277	250,814

Given the highly fragmented nature of the SSH library, most genes are represented by multiple non-overlapping contigs, which makes a comparative quantitative analysis of expression levels problematic. To reduce redundancy, the *de novo* assembled contigs were therefore further assembled by mapping to reference sequences.

The contigs were first mapped to the *M. truncatula* CDSs (Mt4.0v1_GenesCDSSeq_20130731_1800.fasta). Consensus sequences were extracted inserting “N” for ambiguity symbols and IUPAC ambiguity codes to resolve conflicts. This generated a set of 10,413 contigs named SSHrefseqM. The un-mapped contigs were then mapped to the white clover transcriptome (Nagy et al., 2013) and consensus sequences extracted in the same way to generate a set of 3,322 contigs named SSHrefseqW. The remaining un-mapped contigs (2,458) were named SSHrefseqA. The reference-based assembly was carried out using a mismatch cost of 2, insertion and deletion costs of 3, length fraction of 0.5 and similarity fraction of 0.8. The three sets were combined in a single reference transcriptome of 16,193 contigs of average length 625 bp named SSHrefseqAMW (Data Sheet 1 of Supplemental Data). A summary of the *de novo* and reference-based assembly is shown in Tables 2A,B, respectively.

Table 2**Summary of *de novo* (A) and reference-based assembly (B)**.

**Table:** 

**(A)**	
Total number of input reads	50,756,845
Total number of *de novo* contigs	20,981
Average contig length in bp	482
N50	546
Number of mapped reads	35,823,839
Average length of mapped reads in bp	110
Mean base coverage	387x

**Table:** 

**(B)**			
	**Count**	**Average length**	**No. of bases**
Reference transcriptome (*M. truncatula*)	62,319	1,060	66,028,174
No. input contigs	20,981	482	10,119,208
No. mapped contigs	14,785	503	7,442,262
No. un-mapped contigs	6,196	432	2,676,946
No. mapped contig consensuses (SSHrefseqM)	10,413^*^	715	7,442,262
References transcriptome (*T. repens*)	71,545	563	40,246,931
No. input contigs	6,196	432	2,676,946
No. mapped contigs	3,738	416	1,554,812
No. un-mapped contigs (SSHrefseqA)	2,458^*^	457	1,122,134
No. mapped contig consensuses (SSHrefseqW)	3,322^*^	468	1,554,812
SSHrefseqAMW transcriptome reference	**16,193**	625	10,119,208

*The total number of contigs in SSHrefseqAMW (in bold) is the sum of the consensus contigs (marked with an asterisk) mapped to M. truncatula (SSHrefseqM), T. repens (SSHrefseqW) and un-mapped (SSHrefseqA). Mean base coverage was calculated as (number of reads mapped X average read length) / total length of contigs*.

The SSHrefseqAMW transcriptome was functionally annotated using Blast2GO (Conesa et al., 2005) (Table S2). In total 78% of the contigs had BLASTx hits in the NCBI database and 65% could be annotated. The great majority of the top BLAST hits were from *M. truncatula* and *Cicer arietinum* (43%), followed by *Glycine max* and *Phaseolus vulgaris* (6 and 3%, respectively).

### Mapping of the four SSH libraries to SSHrefseqAMW

The reads from the four SSH libraries DF, DR, RF, and RR were independently mapped to the newly generated reference transcriptome SSHrefseqAMW using the same parameters as above, except for the similarity fraction, which was increased to 0.9. Table 3 shows a summary of the results from this mapping.

**Table 3:** **Summary of the library-specific read mapping to the SSHrefseqAMW transcriptome reference**.

	**Counts**	**% of reads**	**Average length**	**Number of bases**
SSHrefseqAMW	16,193	-	625.00	10,119,208.00
DF mapped reads	3,151,998	61.39	102.07	321,718,463.00
DF contigs	11,095	-	310.00	3,437,391.00
DR mapped reads	14,041,207	76.33	110.40	1,550,159,323.00
DR contigs	10,118	-	315.00	3,187,663.00
RF mapped reads	14,975,129	69.28	113.87	1,705,150,875.00
RF contigs	15,829	-	467.00	7,385,140.00
RR mapped reads	3,511,609	62.58	105.10	369,084,303.00
RR contigs	9,387	-	291.00	2,732,913.00

**Figure 4:**
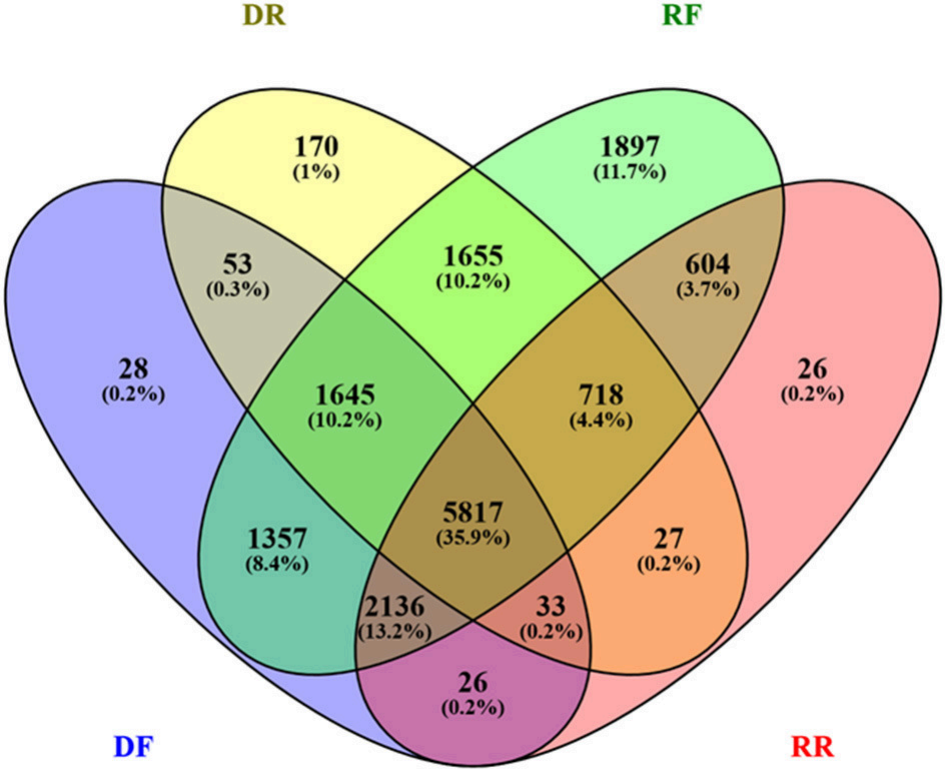
**Venn diagram showing the extent of redundancy between libraries**. The number of contigs in each library is expressed as absolute value and percent (in brackets).

A considerable overlapping between the four libraries was found (Figure 4). The SSH procedure is known to produce a number of false positives. The SSH procedure is based on two rounds of hybridisation and PCR amplification, with the rate of hybridization depending heavily on the transcript abundance, the degree of differential expression and the transcript length. Most genes are differentially regulated from a basal level, rather than being switched on and off, with the majority displaying limited differential expression. With a mathematical model (Gadgil et al., 2002), showed that under recommended conditions the SSH procedure is biased toward transcripts differentially regulated to a large degree and that most false-positives are contributed by rare sequences that are not differentially expressed. Furthermore, Bui et al. (2005) showed experimentally that abundant transcript may escape both subtraction and normalization. A major source of contamination may also come from the non-specific hybridisation between strands of partially homologous genes (Gadgil et al., 2002). While the effect of these factors could be trivial when the sequencing is carried out on a limited number of bacterial clones, it becomes significant if the SSH procedure is followed by deep sequencing, which in this case yielded a base coverage of nearly 400x (Table 2A). To resolve this contamination, expression levels in RPKM (Reads Per Kilobase per Million; Mortazavi et al., 2008) were analyzed. First contigs shorter than 50 bp were filtered out. Contigs shared between forward and reverse drought libraries were also discarded if the difference in RPKM was not significant (cut off of *q* = 0.05). The remaining contigs were assigned to the forward library if the log_2_ fold change ≥ 2 or to the reverse if ≥ −2. The DF library built up in this way was then subtracted *in silico* from the RF library and the remaining contigs were compared to the RR library in the same way as DF/DR comparison.

Three sets of putative differentially expressed genes were generated. The first set comprises all contigs mapped in each library (non-Subtracted set, nS); the second set comprises only contigs unique to forward or reverse library (Subtracted and non-Enriched set, SnE); the third set comprises the subtracted contigs enriched for contigs with log_2_ fold change ≥ ±2 (Subtracted and Enriched set, SE). Results are summarized in Table 4 and full data set can be found in Table S3.

**Table 4:** **Total number of contigs ≥50 bp in each library set and results of the functional annotation of individual libraries**.

	**Total No. of contigs**	**Contigs with BLAST hits %**	**Contigs with annotation %**	**Contigs assigned to KEGG pathway**
**nS**				
DF	9,587	81.89	69.13	140
DR	8,124	83.01	72.07	115
RF	15,665	78.74	65.11	142
RR	7,477	81.82	69.18	134
**SnE**				
DF	4,077	76.80	60.95	116
DR	2,614	77.43	65.49	115
RF	6,528	75.54	61.35	129
RR	2,325	76.77	60.69	106
**SE**				
DF	6,937	80.39	65.93	135
DR	3,142	79.25	68.95	120
RF	6,695	76.25	62.79	127
RR	4,897	80.84	66.71	128

*nS, non-Subtracted; SnE, Subtracted non-Enriched (i.e., unique to each library); SE, Subtracted and log2-fold change Enriched*.

### Functional annotation by Go terms and KEGG pathways

Contigs in each sets were compared for their enrichment in GO (Gene Ontology) terms and KEGG (Kyoto Encyclopedia of Genes and Genomes) pathways representation using Blast2GO.

In each library BLASTx hits were found for 75–83% of the contigs and functional annotations could be assigned to 61–72% of the contigs (Table 4).

**Figure 5:**
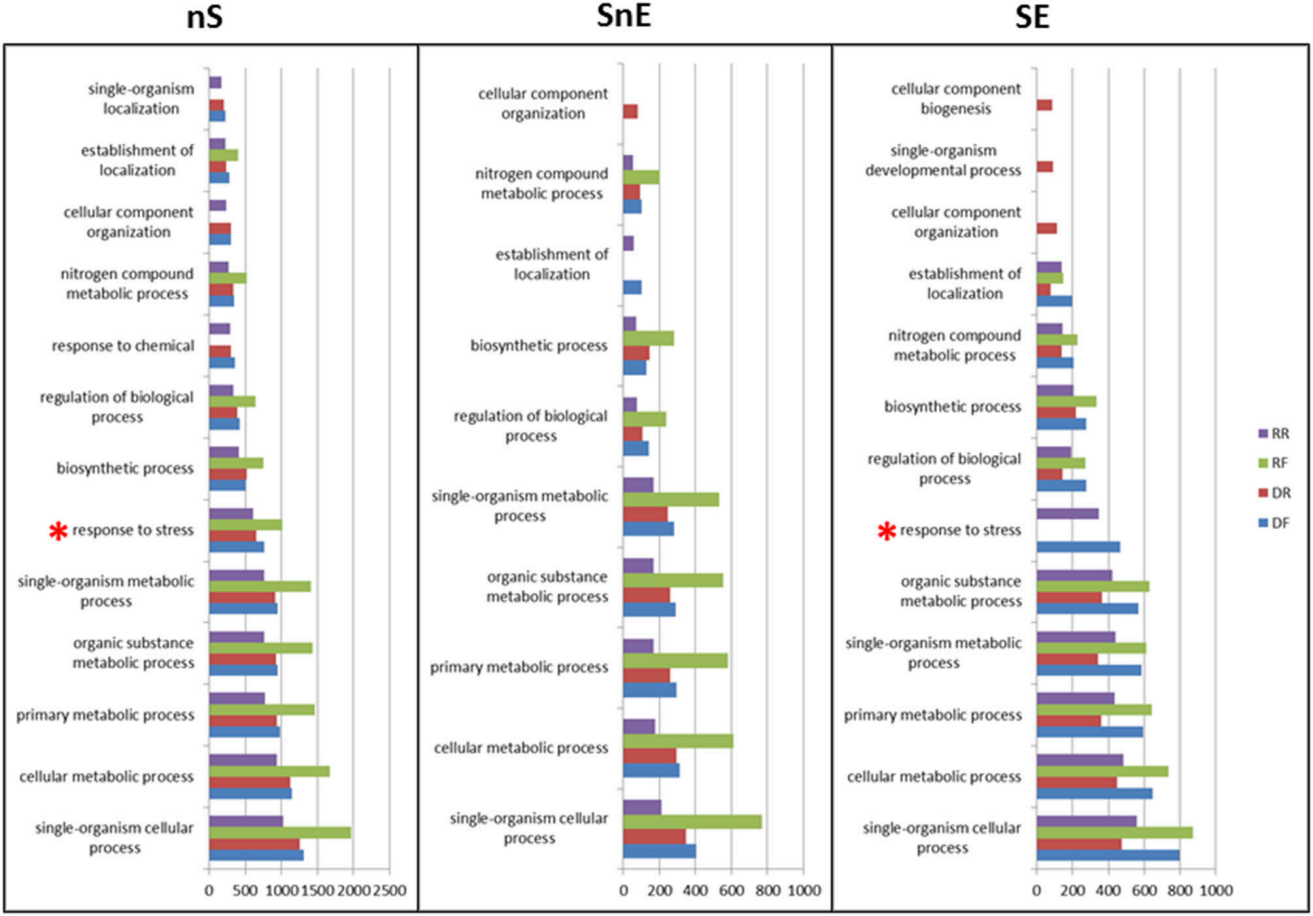
**GO term assignment in the biological process category**. Go terms at levels 2 and 3 are shown in the non-Subtracted (nS), Subtracted non-Enriched (SnE), and Subtracted Enriched (SE) sets. Values on the x axis are scores computed by Blast2GO as per Conesa et al. (2005).

Go terms associated with each library in the Biological Process category where compared in the three sets. Figure 5 shows the results for level 3 using a node score filter of 10% (full data available in Table S4). In the nS set all libraries appear to be similarly enriched in the GO term of this category. With the exception of the “response to chemical,” “cellular component organization” and “single-organism localization” that are not represented in the RF library, all other terms appear to be over-represented and following the same pattern. When the *in silico* subtraction and/or enrichment are applied (SnE and SE sets) some GO terms appear to be more specific to some libraries. In particular the “response to stress” term (marked with a red asterisk), which is expected to be over-represented in a library of material subjected to drought, shows the expected pattern only in the SE set: present in DF (up-regulated under drought) and in RR (down-regulated during recovery).

As part of the Blast2GO analysis contigs in each library were assigned with an EC (Enzyme Code) number and mapped to the relevant KEGG pathway. Contigs were assigned to 106–142 pathways depending on the size of the library (Table 4). Tables 5, 6 summarize the results of the KEGG analysis for the drought and recovery library, respectively. Only pathways significantly enriched (*p* < 0.05), showing at least 50% of the number of enzymes present in the equivalent pathway of *M. truncatula* (mtr) and at least 1.5 folds difference between forward and reverse library are shown (full set of data available in Table S5). As expected, the largest number of differentially expressed contigs was associated with metabolic processes, such as amino acid, carbohydrate, nucleotide and lipid metabolism. All pathways appear to be over-represented in both drought and recovery forward libraries of the non-subtracted set (marked in bold), including pathways that are well-known to be downregulated under drought, such as those related to translation and energy metabolism. Those pathways are indeed under-represented in both SnE and SE sets from the drought treatment (Table 5) and over-represented in all sets (energy metabolism) or just in the SnE and SE sets (translation) from the recovery treatment (Table 6).

**Table 5:** **List of KEGG pathways represented in each set as involved in response to drought**.

**Category**	**nS**				**SnE**				**SE**			
	**Pathway**	**DF**	**DR**	**mtr**	**Pathway**	**DF**	**DR**	**mtr**	**Pathway**	**DF**	**DR**	**mtr**
Amino acid metabolism	**Cysteine and methionine metabolism**	29	16	28	**Valine, leucine and isoleucine degradation**	12	5	18	**Arginine and proline metabolism**	15	7	22
	**Glycine, serine and threonine metabolism**	21	14	31	**Arginine and proline metabolism**	12	5	22	**Valine, leucine and isoleucine degradation**	13	6	18
	**Phenylalanine metabolism**	18	12	15	**Tyrosine metabolism**	9	5	13	**Tyrosine metabolism**	11	5	13
	**Alanine, aspartate and glutamate metabolism**	17	10	19	**Lysine degradation**	8	3	15	**Arginine biosynthesis**	11	5	14
	**Arginine and proline metabolism**	17	8	22	Lysine biosynthesis	3	5	8	**Lysine degradation**	9	3	15
	**Valine, leucine and isoleucine degradation**	14	9	18								
	**Tyrosine metabolism**	13	8	13								
	**Arginine biosynthesis**	13	7	14								
	**Lysine degradation**	9	4	15								
	**Lysine biosynthesis**	8	4	8								
	**Valine, leucine and isoleucine biosynthesis**	7	3	9								
Carbohydrate metabolism	**Amino sugar and nucleotide sugar metabolism**	33	16	37	**Pentose phosphate pathway**	12	5	14	**Galactose metabolism**	13	6	15
	**Starch and sucrose metabolism**	32	20	33	**Galactose metabolism**	12	6	15	**Propanoate metabolism**	12	2	15
	**Glyoxylate and dicarboxylate metabolism**	22	13	14	**Propanoate metabolism**	8	2	15	**Citrate cycle (TCA cycle)**	11	7	16
	**Glycolysis / Gluconeogenesis**	22	14	25	Pyruvate metabolism	6	11	21	**Pentose and glucuronate interconversions**	10	5	11
	**Pentose phosphate pathway**	16	9	14	Ascorbate and aldarate metabolism	3	6	12	**Butanoate metabolism**	10	3	12
	**Fructose and mannose metabolism**	16	10	23								
	**Galactose metabolism**	15	8	15								
	**Inositol phosphate metabolism**	15	8	21								
	**Propanoate metabolism**	14	5	15								
	**Citrate cycle (TCA cycle)**	14	8	16								
	**Pentose and glucuronate interconversions**	12	7	11								
	**Butanoate metabolism**	12	7	12								
	**Ascorbate and aldarate metabolism**	11	4	12								
Nucleotide metabolism	**Purine metabolism**	35	8	39	**Purine metabolism**	21	14	39	**Purine metabolism**	27	14	39
	**Pyrimidine metabolism**	18	6	31					**Pyrimidine metabolism**	16	9	31
Lipid metabolism	**Glycerolipid metabolism**	19	8	18	**Fatty acid degradation**	9	4	9	**Fatty acid degradation**	12	5	9
	**Glycerophospholipid metabolism**	19	8	29	**Fatty acid elongation**	6	0	10	**Sphingolipid metabolism**	9	4	16
	**Fatty acid degradation**	12	6	9	Glycerolipid metabolism	6	11	18	**Fatty acid elongation**	6	1	10
	**Sphingolipid metabolism**	10	1	16	Synthesis and degradation of ketone bodies	1	2	3	**Arachidonic acid metabolism**	5	1	8
	**Steroid biosynthesis**	8	4	11					**Linoleic acid metabolism**	3	2	5
	**Fatty acid biosynthesis**	7	4	13								
	**Arachidonic acid metabolism**	6	3	8								
	**Fatty acid elongation**	6	3	10								
Metabolism of cofactors and vitamins	**Pantothenate and CoA biosynthesis**	12	5	14	**Pantothenate and CoA biosynthesis**	8	2	14	**Pantothenate and CoA biosynthesis**	11	3	14
	**One carbon pool by folate**	8	1	8	**Nicotinate and nicotinamide metabolism**	5	2	10	**Nicotinate and nicotinamide metabolism**	7	2	10
	**Nicotinate and nicotinamide metabolism**	8	1	10	Porphyrin and chlorophyll metabolism	4	13	25	**Vitamin B6 metabolism**	4	2	8
	**Riboflavin metabolism**	5	1	8					**Thiamine metabolism**	4	2	8
	**Thiamine metabolism**	4	1	8					Porphyrin and chlorophyll metabolism	8	17	25
Metabolism of other amino acids	**beta-Alanine metabolism**	12	7	14	**beta-Alanine metabolism**	9	3	14	**beta-Alanine metabolism**	12	3	14
	**Selenocompound metabolism**	8	3	8	Cyanoamino acid metabolism	1	4	6	**Taurine and hypotaurine metabolism**	2	1	3
	**Cyanoamino acid metabolism**	7	2	6	Selenocompound metabolism	2	4	8				
Biosynthesis of other secondary metabolites	**Phenylpropanoid biosynthesis**	12	8	18	**Tropane, piperidine and pyridine alkaloid biosynthesis**	6	4	6	**Tropane, piperidine and pyridine alkaloid biosynthesis**	7	4	6
	**Monobactam biosynthesis**	4	1	6	**Caffeine metabolism**	2	1	2	**Caffeine metabolism**	3	1	2
	**Stilbenoid, diarylheptanoid and gingerol biosynthesis**	4	2	7	Monobactam biosynthesis	0	4	6	Flavone and flavonol biosynthesis	1	2	2
					Flavone and flavonol biosynthesis	0	1	2	Isoflavonoid biosynthesis	1	2	3
Translation	**Aminoacyl-tRNA biosynthesis**	18	12	20	Aminoacyl-tRNA biosynthesis	1	18	20	Aminoacyl-tRNA biosynthesis	9	18	20
Energy metabolism	**Carbon fixation in photosynthetic organisms**	21	11	18	Nitrogen metabolism	1	5	8	Carbon fixation in photosynthetic organisms	8	15	18
	**Sulfur metabolism**	13	4	7								
	**Oxidative phosphorylation**	7	2	7								

*Only pathways significantly enriched (p < 0.05), containing at least 50% of the number of enzymes present in the equivalent pathway of M. truncatula (mtr) and with at least 1.5-folds difference between forward and reverse library are shown. Pathways up-regulated under drought are marked in bold*.

**Table 6:** **List of KEGG pathways represented in each set as involved in recovery from drought**.

**Category**	**nS**				**SnE**				**SE**			
	**Pathway**	**RF**	**RR**	**mtr**	**Pathway**	**RF**	**RR**	**mtr**	**Pathway**	**RF**	**RR**	**mtr**
Carbohydrate metabolism	**Ascorbate and aldarate metabolism**	11	7	12	**Starch and sucrose metabolism**	29	12	33	**Glyoxylate and dicarboxylate metabolism**	22	9	22
					**Amino sugar and nucleotide sugar metabolism**	21	6	37	**Pyruvate metabolism**	21	11	21
					**Pyruvate metabolism**	20	5	21	**Glycolysis/Gluconeogenesis**	20	11	20
					**Glyoxylate and dicarboxylate metabolism**	17	4	14	**Fructose and mannose metabolism**	17	10	17
					**Glycolysis/Gluconeogenesis**	16	8	25	Propanoate metabolism	4	11	4
					**Galactose metabolism**	15	8	15				
					**Fructose and mannose metabolism**	15	5	23				
					**Citrate cycle (TCA cycle)**	14	2	16				
					**Butanoate metabolism**	9	2	12				
					**Ascorbate and aldarate metabolism**	6	1	12				
					**C5-Branched dibasic acid metabolism**	2	0	4				
Biosynthesis of other secondary metabolites	**Flavonoid biosynthesis**	12	8	13	**Flavonoid biosynthesis**	8	3	13	**Phenylpropanoid biosynthesis**	10	6	10
	**Isoquinoline alkaloid biosynthesis**	6	4	11	**Isoquinoline alkaloid biosynthesis**	6	4	11	**Flavonoid biosynthesis**	9	6	9
	**Monobactam biosynthesis**	4	2	6	**Monobactam biosynthesis**	4	0	6	**Isoquinoline alkaloid biosynthesis**	6	4	6
	**Stilbenoid, diarylheptanoid and gingerol biosynthesis**	4	2	7	**Flavone and flavonol biosynthesis**	2	0	2	**Monobactam biosynthesis**	4	0	4
	**Flavone and flavonol biosynthesis**	3	2	2					**Flavone and flavonol biosynthesis**	3	0	3
Energy metabolism	**Sulfur metabolism**	15	8	7	**Sulfur metabolism**	11	6	7	**Carbon fixation in photosynthetic organisms**	20	6	20
	**Nitrogen metabolism**	11	6	8	**Carbon fixation in photosynthetic organisms**	10	4	18	**Sulfur metabolism**	12	6	12
Metabolism of cofactors and vitamins	**Porphyrin and chlorophyll metabolism**	24	13	25	**Porphyrin and chlorophyll metabolism**	14	4	25	**Porphyrin and chlorophyll metabolism**	18	7	18
	**Nicotinate and nicotinamide metabolism**	9	6	10	**One carbon pool by folate**	8	2	8	**One carbon pool by folate**	8	4	8
					**Riboflavin metabolism**	4	1	8	Pantothenate and CoA biosynthesis	3	10	3
					Pantothenate and CoA biosynthesis	3	7	14	Thiamine metabolism	2	4	2
Amino acid metabolism	**Phenylalanine, tyrosine and tryptophan biosynthesis**	23	14	20	**Cysteine and methionine metabolism**	19	11	28	**Glycine, serine and threonine metabolism**	18	10	18
	**Alanine, aspartate and glutamate metabolism**	21	13	19	**Phenylalanine, tyrosine and tryptophan biosynthesis**	15	4	20	Valine, leucine and isoleucine degradation	6	13	6
					**Alanine, aspartate and glutamate metabolism**	12	3	19	Lysine degradation	4	8	4
					**Arginine biosynthesis**	8	3	14				
					**Histidine metabolism**	5	3	9				
Nucleotide metabolism	**Purine metabolism**	42	27	39	**Purine metabolism**	27	11	39	**Pyrimidine metabolism**	20	13	20
	**Pyrimidine metabolism**	25	15	31	**Pyrimidine metabolism**	19	6	31				
Metabolism of other amino acids	**Selenocompound metabolism**	10	6	8	**Glutathione metabolism**	10	4	17	**Selenocompound metabolism**	8	3	8
					**Selenocompound metabolism**	8	0	8	beta-Alanine metabolism	6	11	6
Glycan biosynthesis and metabolism	**N-Glycan biosynthesis**	9	4	17	**Cyanoamino acid metabolism**	4	1	6	**Other glycan degradation**	5	3	5
	**Other glycan degradation**	6	4	7	**Taurine and hypotaurine metabolism**	2	1	3	**Glycosaminoglycan degradation**	3	1	3
	**Glycosaminoglycan degradation**	3	1	6	**Zeatin biosynthesis**	3	0	3	**Glycosphingolipid biosynthesis - ganglio series**	2	1	2
	**Glycosphingolipid biosynthesis - ganglio series**	2	1	4					**Glycosphingolipid biosynthesis - globo series**	2	1	2
	**Glycosphingolipid biosynthesis - globo series**	2	1	4								
Lipid metabolism	**Sphingolipid metabolism**	11	6	16	**Glycerolipid metabolism**	13	6	18	Fatty acid degradation	4	12	4
	**Arachidonic acid metabolism**	8	5	8	**Sphingolipid metabolism**	8	4	16	Fatty acid elongation	2	6	2
	**Synthesis and degradation of ketone bodies**	4	2	3	**Fatty acid elongation**	5	2	10	Linoleic acid metabolism	2	3	2
					**Arachidonic acid metabolism**	4	0	8				
					**Linoleic acid metabolism**	3	0	5				
					**Synthesis and degradation of ketone bodies**	2	1	3				
Metabolism of terpenoids and polyketides	**Terpenoid backbone biosynthesis**	19	9	23								
	**Zeatin biosynthesis**	3	0	3								

*Only pathways significantly enriched (p < 0.05), containing at least 50% of the number of enzymes present in the equivalent pathway of M. truncatula (mtr) and with at least 1.5 folds difference between forward and reverse library are shown. Pathways up-regulated during recovery are marked in bold*.

In plants, such as white clover, that employ drought tolerance as the main strategy for coping with stress, the response is primarily focused on maintaining the cell water potential. This is achieved by the accumulation of soluble small molecular weight osmoprotectants, such as amino acids (proline, asparagine, serine), polyamines (putrescine, spermidine, spermine), glycine betaine and γ-amino-N-butyric acid (GABA) (Bartels and Sunkar, 2005). This is in agreement with the “arginine and proline metabolism” pathway, involved in the biosynthesis of proline, putrescine, spermidine and spermine, being one of the most represented in the DF library. It is also in line with the work of Li et al. (2015a,b), which showed the positive effect of both exogenous and endogenous polyamines on white clover tolerance to water deficit. Also significantly over-represented in the DF library is the “butanoate metabolism” pathway, which is the main route of GABA biosynthesis via decarboxylation of L-glutamate.

Regulation of osmotic adjustment can also be achieved via carbohydrates, which are accumulated to varying degrees in different plants (Singh et al., 2015). Glucose, sucrose and fructose are the most common sugars to be accumulated under stress, however raffinose family oligosaccharides (RFOs), such as raffinose, stachyose, and verbascose, have also been shown to play a role in a variety of stress responses (Castonguay and Nadeau, 1998; Taji et al., 2002; Peters et al., 2007; Nishizawa et al., 2008; Egert et al., 2015). The first report of RFOs involvement in the response to drought came from the observed increased tolerance of *Arabidopsis* plants over-producing galactinol synthase, which catalyzes the first step in the synthesis of RFOs (Taji et al., 2002). An increased concentration of RFOs has also been associated with drought tolerance in alfalfa (Kang et al., 2011) and in various resurrection plants (Peters et al., 2007; Egert et al., 2015). It has been suggested that RFOs not only act as osmoprotectants, but also as ROS (Reactive Oxygen Species) scavenger providing protection from oxidative damage (Nishizawa et al., 2008). White clover is known to increase the concentration of soluble sugars under water deficit (Turner, 1990; Lee et al., 2008; Li et al., 2013) but their composition is not known. The over-representation of the “galactose metabolism” pathway (87 and 80% of the enzymes in the equivalent of *M. truncatula* in SnE and SE, respectively) suggests that RFOs play an important role at the onset of white clover response to drought. It has to be noted that other pathways in the “carbohydrate metabolism” category that are related to the accumulation of sugars, such as “starch and sucrose metabolism,” are also significantly over-represented in the DF library (see Table S5), but the difference between DF and DR is <1.5 folds and therefore have not been shown in Table 5. However, it is quite possible these pathways will appear more enriched at later stages of the drought treatment. The “fructose and mannose metabolism” pathway, on the other hand, is non-significantly over-represented in DR compared to DF, but significantly over-represented in RF compared to RR in the SnE and SE sets. It could be extrapolated that, following a delayed activation at a later stage of drought, the expression of the related genes is still elevated at day 1 of recovery (Table 6 and Table S5).

It is well-known that inhibition of photosynthesis is among the early effects of drought stress. This is reflected in the down-regulation of genes encoding enzymes related to the “carbon fixation in photosynthetic organisms” and “porphyrin and chlorophyll metabolism” pathways (83 and 68% of the enzymes in the equivalent pathways of *M. truncatula*, respectively, in the SE set). As expected, these pathways are enriched in the RF library compared to DR in both SnE and SE set (Table 7). Also downregulated under drought treatment are 90% of the genes encoding enzymes related to the “aminoacyl-tRNA biosynthesis” pathways. This trend has been observed in other plants (Yamakawa and Hakata, 2010; Merewitz et al., 2011; Mohanty et al., 2016) and it has been related to the aminoacyl-tRNA molecules being associated with other processes in addition to protein synthesis, such as synthesis of porphyrin ring structure (Mocibob et al., 2010), and to the accumulation of osmolytes in the form of free amino acids (Yamakawa and Hakata, 2010).

Table 7**Comparison between RPKM values (A) and sq-RT-PCR results (B)**.

**Table:** 

**(A)**																
**Contig**	**RPKM**				**DATA set**											
	**DF**	**DR**	**RF**	**RR**	**nS**				**SnE**				**SE**			
SSHrefM3_contig_8638	0.00	6.93	0.95	0.00	–	DR	RF	–	–	DR	RF	–	–	DR	RF	–
SSHrefM3_contig_9592	0.00	72.41	2.55	0.00	–	DR	RF	–	–	DR	RF	–	–	DR	RF	–
SSHrefA3_contig_507	1.83	0.00	28.37	6.04	DF	–	RF	RR	DF	–	rf	RR	DF	–	rf	RR
SSHrefM3_contig_698	1.78	0.00	2.08	1.75	DF	–	RF	RR	DF	–	rf	RR	DF	–	rf	RR
SSHrefM3_contig_7860	3.75	0.00	0.16	2.02	DF	–	RF	RR	DF	–	rf	RR	DF	–	rf	RR
SSHrefM3_contig_1729	4.08	38.65	30.9	4.44	DF	DR	RF	RR	df	dr	rf	rr	df	DR	RF	rr
SSHrefM3_contig_2464^†^	6.84	72.93	50.44	5.03	DF	DR	RF	RR	df	dr	rf	rr	df	DR	RF	rr
SSHrefM3_contig_7685	14.12	0.00	1.83	12.68	DF	–	RF	RR	DF	–	rf	RR	DF	–	rf	RR
SSHrefM3_contig_6945	18.83	0.00	1.22	5.20	DF	–	RF	RR	DF	–	rf	RR	DF	–	rf	RR
SSHrefM3_contig_564^†^	24.3	124.5	858.03	32.24	DF	DR	RF	RR	df	dr	rf	rr	df	DR	RF	rr
SSHrefW3_contig_1414^*^	26.38	3.18	45.65	95.31	DF	DR	RF	RR	df	dr	rf	rr	DF	dr	rf	RR
SSHrefM3_contig_2299^*^	42.16	0.71	38.99	75.21	DF	DR	RF	RR	df	dr	rf	rr	DF	dr	rf	RR
SSHrefM3_contig_3336^*^	48.95	0.81	31.29	120.42	DF	DR	RF	RR	df	dr	rf	rr	DF	dr	rf	RR
SSHrefA3_contig_859^†^	517.36	0.00	24.8	336.76	DF	–	RF	RR	DF	–	rf	RR	DF	–	rf	RR
SSHrefM3_contig_3263^*^	734.40	5.33	427.96	824.25	DF	DR	RF	RR	df	dr	rf	rr	DF	dr	rf	RR
SSHrefW3_contig_2289^*^	1803.00	9.89	625.81	2253.85	DF	DR	RF	RR	df	dr	rf	rr	DF	dr	rf	RR
SSHrefM3_contig_3795^*^	3441.00	39.97	1314.08	3279.21	DF	DR	RF	RR	df	dr	rf	rr	DF	dr	rf	RR
SSHrefM3_contig_3892^*^	8341.00	0.21	380.12	6350.87	DF	DR	RF	RR	df	dr	rf	rr	DF	dr	rf	RR
SSHrefM3_contig_611^*^	34441.00	194.20	44141.60	40870.28	DF	DR	RF	RR	df	dr	rf	rr	DF	dr	rf	RR
SSHrefM3_contig_329^†^	71963.76	16.78	18516.37	64957.88	DF	DR	RF	RR	df	dr	rf	rr	DF	dr	rf	RR

**Table:** 

**(B)**					
**Contig**	**Function**	**C**	**Day D4**	**Day D9**	**Day R1**
SSHrefM3_contig_8638	proline dehydrogenase mitochondrial-like (EC:1.5.5.2)	1	1.06±0.17	0.56±0.13	9.26±0.35
SSHrefM3_contig_9592	uncharacterized calcium-binding protein at1g02270-like	1	0.68±0.21	1.13±0.21	1.7±0.34
SSHrefA3_contig_507	alpha beta hydrolase domain-containing protein 11-like	1	2.69±0.61	2.01±0.32	1.17±0.65
SSHrefM3_contig_698	abc transporter b family member 19-like (EC:3.6.3.44)	1	1.63±0.29	2.17±0.29	0.41±0.21
SSHrefM3_contig_7860	formate–tetrahydrofolate ligase-like (EC:6.3.4.3)	1	2.68±0.28	1.58±0.40	1.36±0.23
SSHrefM3_contig_1729	caffeic acid 3-o-methyltransferase-like (EC:2.1.1.76)	1	3.61±0.14	7.52±0.18	2.07±0.41
SSHrefM3_contig_2464^†^	heat shock cognate protein 80-like	-	−	−	−
SSHrefM3_contig_7685	isoflavone reductase homolog (EC:1.3.1.45)	1	4.67±0.38	2.3±0.41	0.84±0.42
SSHrefM3_contig_6945	acid beta-fructofuranosidase-like (EC:3.2.1.48, EC:3.2.1.26, EC:3.2.1.80)	1	1.88±0.28	7.39±0.2	1.05±0.62
SSHrefM3_contig_564^†^	ubiquitin 11	-	−	−	−
SSHrefW3_contig_1414^*^	6-phosphogluconate decarboxylating 3 (EC:1.1.1.44)	1	4.47±0.2	3.82±0.56	0.71±0.27
SSHrefM3_contig_2299^*^	aconitate hydratase 1 (EC:4.2.1.3)	1	3.15±0.41	3.54±0.34	0.57±0.21
SSHrefM3_contig_3336^*^	bifunctional nuclease 1	1	22.48±0.43	16.68±0.4	0.38±0.26
SSHrefA3_contig_859^†^	unknown	-	−	−	−
SSHrefM3_contig_3263^*^	26s proteasome non-atpase regulatory subunit rpn12a-like	1	12.77±0.58	3.1±0.4	1.75±0.36
SSHrefW3_contig_2289^*^	cinnamoyl- reductase 1-like	1	3.67±0.17	5.23±0.16	0.74±0.32
SSHrefM3_contig_3795^*^	pathogenesis-related protein pr-4-like	1	53.32±0.39	274.31±0.41	13.98±0.43
SSHrefM3_contig_3892^*^	universal stress protein	1	5.92±0.67	40.38±0.2	1.05±0.41
SSHrefM3_contig_611^*^	thiol protease aleurain-like	1	7.15±0.38	12.01±0.97	0.44±0.72
SSHrefM3_contig_329^†^	abscisic acid stress ripening protein	-	−	−	−

*In each library (–) denotes absence of the contig, small case denotes loss of the contig following statistical analysis; capital letter denotes retaining of the contig following statistical analysis. Contigs failed to amplify in sqRT-PCR are marked with a cross (^†^)*.

Contigs rescued by the enrichment of the SnE set are marked with an asterisk (^*^). Relative expression was measured at day 4 and 9 of drought and day 1 of recovery as compared to day 1 of the experiment (C). Actin was used as an internal control. Results are expressed as means ± standard deviation (n = 3).

It was however unexpected to find a higher representation of the “flavone and flavonol biosynthesis” and “isoflavonoid biosynthesis” pathways in the DR library, and non-significant difference between the DF and DR libraries in the enrichment of the “flavonoid biosynthesis” pathway (Table S5). Flavonoids are secondary metabolites with strong antioxidant activity that are involved in plant protection against biotic and abiotic stresses, including drought (Tattini et al., 2004; Mouradov and Spangenberg, 2014). Flavonoids are classified in several sub-groups, one of which, isoflavonoids, is predominantly found in leguminous plants. (Iso) flavonoids have been shown to accumulate in shoots of alfalfa and leaves of *Lotus japonicus* (Kang et al., 2011; Garcia-Calderon et al., 2015). Ballizany et al. (2012b) showed an increase of flavonol glycosides of quercetin in white clover subject to water deficit and its positive association with retention of higher level of dry matter production under stress. In our study the pathways related to flavonoid biosynthesis are all significantly over-represented in the DF library (Table S5), but not compared to the DR library. In Ballizany et al. (2012b), the water deficit imposed was much harsher then in our experiment (9, 13, and 17 weeks above wilting point). Our library was constructed from material at the onset of the drought response and it is therefore conceivable that the biosynthesis of flavonoid is also at an early stage. In agreement with this hypothesis is the enrichment of these pathways in all three sets of the recovery treatment (Table 6). As hypothesized above for the “fructose and mannose metabolism,” transcripts associated with the synthesis of flavonoids could still be abundant following activation during advanced stages of drought stress. A time course of the expression of the genes involved in the different branches of the phenolic biosynthetic pathway in white clover under drought stress will help characterizing the stress response in more details and will clarify the potential of flavonoids as a target for improving drought tolerance (Kang et al., 2011; Ballizany et al., 2012a,b).

### Mapman functional annotation

**Figure 6:**
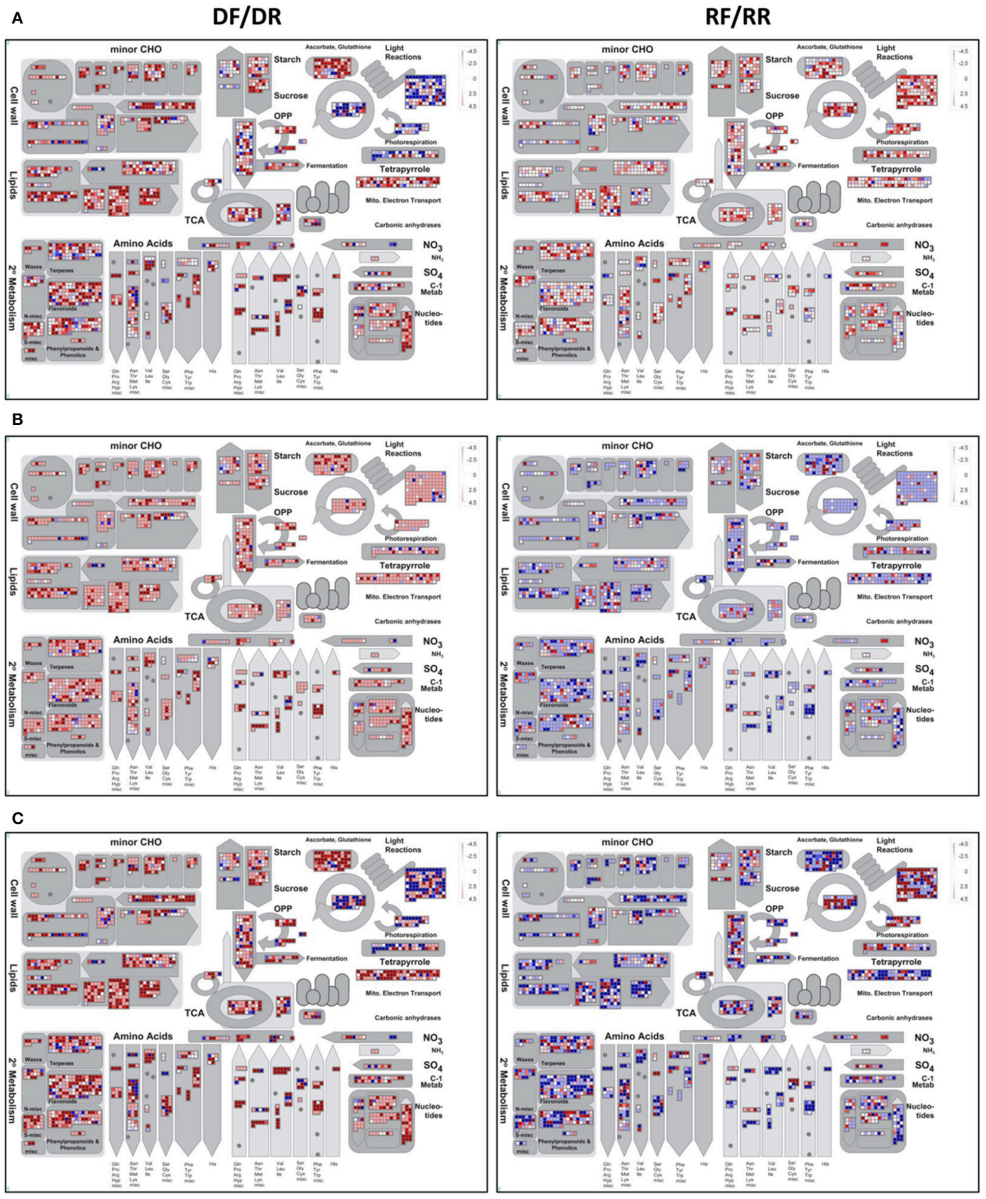
**MapMan metabolic overview of white clover gene expression at the onset of drought (DF/DR) and recovery (RF/RR) in the nS (A)**, SnE **(B)**, and SE **(C)** sets. Each square represents a transcript whose expression can be the same (white square), up-regulated (red squares) or down-regulated (blue squares) in the forward as compared to the reverse libraries.

The differentially expressed contigs were also functionally annotated using MapMan (Thimm et al., 2004). Unlike GO, MapMan has the advantage of being developed for plant-specific pathways and of not separating different functional categories, allowing a global overview of high-throughput data in the context of pathways and processes. MapMan uses a different approach from GO in that it assigns genes to functional categories (BINs), rather than to a GO terms, that are represented in a hierarchically structured tree. For this analysis MapMan bins were assigned to the SSHrefseqAMW reference transcriptome using the Mercator pipeline (Lohse et al., 2014), so that it could be used as a mapping file. The data files were generated by calculating the log_2_ fold change of expression levels (in RPKM) in forward and reverse libraries. A metabolic overview of nS, SnE, and SE set is shown in Figure 6.

The effect of the *in silico* subtraction and/or enrichment is clearly shown by the different patterns of expression in the nS (Figure 6A), SnE (Figure 6B), and SE (Figure 6C) sets. The nS set shows an elevated number of transcripts whose level does not significantly (*p* ≥ 0.05) change between forward and reverse library (white squares), especially in the recovery samples. In the SnE set almost all transcripts appear to be up-regulated (red squares) under drought and down-regulated (blue squares) during recovery. On the other end, the SE set shows a biologically meaningful pattern in which processes related to photosynthesis, such as components of the photosystem (“Light reaction”) and the biosynthesis of photosynthetic pigments (“Tetrapyrrole”) are mostly down-regulated under drought and up-regulated during recovery (Figure 6C). As the rate of photosynthesis slows down the cellular energy is provided by glycolysis and TCA cycle (Fernie et al., 2004) which appear to be up-regulated and down-regulated in drought and recovery, respectively. As discussed in the previous section, carbohydrate and amino acids metabolism are also oppositely regulated under the two treatments. Notably, the flavonoids and phenylpropanoids pathways show an overall up-regulation under water deficit, suggesting that the hierarchical bin system strategy adopted by MapMan is more likely to provide a more accurate representation. Also significantly up- and down-regulated under drought and recovery, respectively, are the transcripts related to the ascorbate-glutathione pathway, involved in ROS scavenging, to cell wall metabolism and to lipids biosynthesis and degradation. Lipids are important components of biological membranes, the initial site of cellular perception of the signals and major targets of environmental stresses. The change in lipids composition in response to stress is well documented (Gigon et al., 2004; Torres-Franklin et al., 2007; Toumi et al., 2008) and plays a primary role not only in the sensing and triggering of the signaling cascade (Wang, 2004) but also in maintaining membrane integrity and preserve cell compartmentalisation.

### Validation of expression profiles by sqRT-PCR

To validate the data obtained from the statistical analysis of the SSH libraries, we carried out a sqRT-PCR. Twenty contigs were semi-randomly chosen to cover low, medium, and high level of expression (in RPKM) in the DF library. The sqRT-PCR data from 16 of the contigs that were successfully amplified validated the expression data obtained from the SSH libraries (Table 7). With the exception of SSHrefM3_contig_1729, whose RT-PCR fit only the nS data set, all the other results fit the dataset SE best. This means that when the sqRT-PCR values are higher or lower than the internal control C on Day D4 (Table 7B), the contig is included in the DF or DR libraries of the SE set, respectively (Table 7A). The same results are observed with the values of Day R1 and the RF or RR libraries. In particular, half of the contigs (marked with an asterisk) with RPKM values ranging from medium to high, would have been lost if the enrichment of the subtracted libraries had not been carried out.

Incidentally, the “false negative” contig, SSHrefM3_contig_1729, is an enzyme from the “Flavone and flavonol biosynthesis” pathway and its expression appears to increase at day 9 of the stress treatment, as hypothesized above.

## Conclusions

In this work we generated the first large scale molecular data related to white clover early response to drought and re-hydration by deep sequencing of SSH libraries. In a previous study only the forward libraries were sequenced using the standard protocol, generating 1,127 and 2,405 sequences for drought and recovery, respectively (Bisaga, 2013). By replacing the cloning step with a MiSeq sequencing run we were able to increase the number of transcripts to 9,587 and 15,665, respectively (Table 4; nS set). Reverse libraries were also sequenced, generating 8,124 and 7,477 transcripts down-regulated during drought and recovery, respectively (Table 4; nS set). These sequences were further processed using a procedure involving *in silico* subtraction of forward and reverse libraries, followed by enrichment with transcripts whose expression level is significantly different in the two libraries. When the three sets of data, non-subtracted (nS), subtracted non-enriched (SnE) and subtracted enriched (SE), were compared by functional annotation only the latter fit all three mapping approaches used, GO term, KEGG pathways and MapMan BINs assignment.

The DF and DR libraries of the SE set, however, are not only quantitatively, but also qualitatively different from the sets of transcripts identified in the original study. About 25% of the transcripts sequenced in the original drought library were not found in the DF library of the SE set. A good example is a set of transcripts associated with the GO term “photosynthesis” (GO:0015979; GO:0019684), which in our study were recovered in every library, and after SE processing, in the DR and RF libraries. This type of artifact has been observed in similar studies, where the same transcript was recovered in both forward and reverse library (Bui et al., 2005; Norelli et al., 2009; Hall et al., 2011). It is believed to be associated with abundant transcript (such as photosynthetic genes), which appear to escape both subtraction and normalization (Bui et al., 2005).

The original recovery forward library was found to be more similar to the one in this study, with only 10% of the transcripts being recovered in libraries different from SE-RF. One example is a set of genes associated with the GO term “response to osmotic stress” (GO:0006970), which in our study was found, as it would be expected, in the DF and RR libraries. In this case, however, only few transcripts appeared to have a high level of expression. The cause of this artifact is therefore different from the one observed for the photosynthetic genes recovered in the drought library, but it is difficult interpret without suitable kinetic studies.

Overall the newly generated sets of data provide a biologically meaningful overview of the changes in gene expression associated with the early response of white clover to drought and rehydration. It provides a valuable resource for candidate genes and molecular markers discovery for improving the crop performance and persistence during the short intermittent drought spells most likely to affect cultivated white clover.

## Author contributions

MB participated in planning the study, carried out the fast drought experiment, the SSH procedure and the libraries construction. ML helped with the fast drought experiment. MH carried out the sequencing and helped with the analysis. MA participated in planning the study. AR conceived and planned the study, directed the work, carried out the analyses and carried out the writing of the manuscript.

## Funding

This work was funded by BBSRC as part of the Institute Strategic Programme Grant.

### Conflict of interest statement

The authors declare that the research was conducted in the absence of any commercial or financial relationships that could be construed as a potential conflict of interest.

## Supplementary material

Image 1 **Pot weight changes during water withdrawal for drought and control plants**.

Table S1 **List of primers used in this study**.

Table S2 **Blast2Go annotation table for the reference transcriptome SSHrefseqAMW**.

Table S3 **List of contigs included in libraries DF, DR, RF, and RR for each data set (nS, SnE, and SE)**.

Table S4 **Blast2Go GO term assignment to libraries DF, DR, RF, and RR for each data set (nS, SnE, and SE)**.

Table S5 **EC2KEGG table of KEGG pathways associated with enzyme codes in libraries DF, DR, RF, and RR for each data set (nS, SnE, and SE)**. Total(EC_All) = number of ECs associated with the KEGG pathway; Total(EC_Ref(mtr)) = number of ECs in reference genome mtr (*M. truncatula*) associated with the KEGG pathway; Total(EC_Given) = number of tested ECs found to be associated with the KEGG pathway; Total(EC_Shared) = number of tested ECs that are shared with reference genome; Total(EC_Unique_Ref) = number of ECs that are unique to the reference genome; Total(EC_Unique_Given) = number of ECs that are unique to the tested genome.
