# Supplementary figures and images for: Deep Sequencing of Suppression Subtractive Hybridisation Drought and Recovery Libraries of the Non-model Crop Trifolium repens L
Source: Front Plant Sci. 2017 Feb 23;8:213. doi: 10.3389/fpls.2017.00213 (PMC5322231; doi:10.3389/fpls.2017.00213)

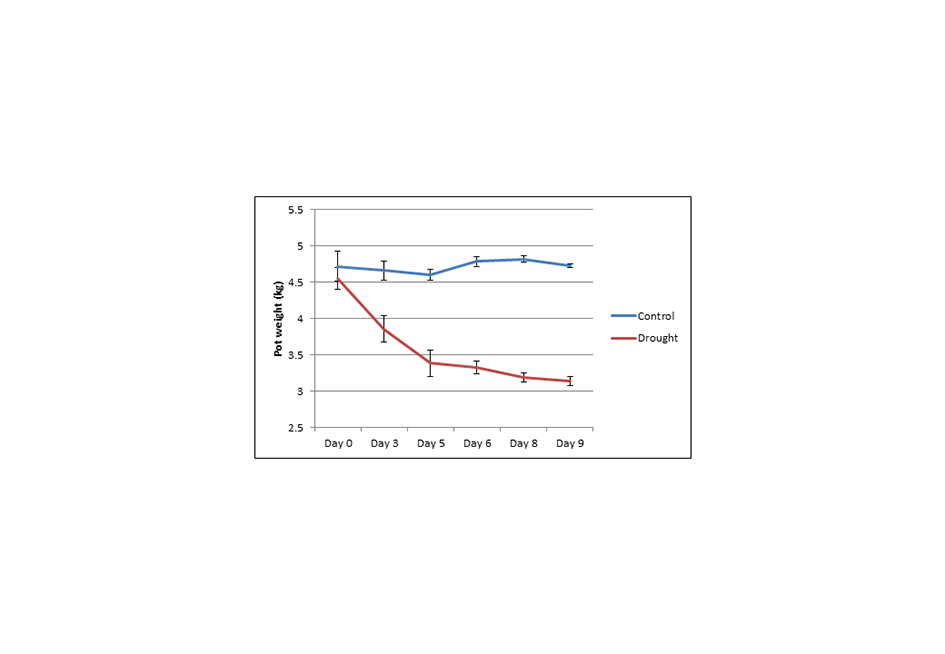

Supplement: Image 1 — Pot weight changes during water withdrawal for drought and control plants. [file Image1.TIF]
